# Phytochemical characterisation of Phlomis linearis Boiss. & Bal and screening for anticholinesterase, antiamylase, antimicrobial, and cytotoxic properties

**DOI:** 10.3906/kim-2009-59

**Published:** 2021-04-28

**Authors:** Gamze GÖGER, Ümmühan TÜRKYOLU, Ezgi Nur GÜRŞEN, Süleyman YUR, Abdullah Burak KARADUMAN, Fatih GÖGER, Mehmet TEKİN, Gülmira ÖZEK

**Affiliations:** 1 Department of Pharmacognosy, Faculty of Pharmacy, Trakya University, Edirne Turkey; 2 Faculty of Pharmacy, Trakya University, Edirne Turkey; 3 Medicinal Plant, Drug and Scientific Research Center (AUBIBAM), Anadolu University, Eskişehir Turkey; 4 Department of Pharmaceutical Toxicology, Faculty of Pharmacy, Anadolu University, Eskişehir Turkey; 5 Department of Pharmacognosy, Faculty of Pharmacy, Anadolu University, Eskişehir Turkey; 6 Department of Pharmaceutical Botany, Faculty of Pharmacy, Trakya University, Edirne Turkey

**Keywords:** *Phlomis linearis*, essential oil, extract, liquid chromatography-mass spectromtetry (LC-MS/MS), gas chromatography-mass spectrometry/flame ionisation detector (GC-MS/FID), activity

## Abstract

In the present work, essential oil and fatty acids and extracts obtained from aerial parts of *Phlomis linearis* Boiss. & Bal. were investigated for chemical composition and biological activities. The phytochemical analyses were conducted with gas chromatography-mass spectrometry/flame ionisation detector (GC-MS/FID) and liquid chromatography-mass spectromtetry (LC-MS/MS) techniques. The extracts and essential oil were studied for *α*-amylase and acetylcholinesterase activities with two different spectrophotometric methods. Antimicrobial activities of the extracts were investigated by microdilution. The extracts were evaluated *in vitro* for cytotoxic effects against cancer and normal cell lines by MTT assay. The essential oil (EO) contained *α*-pinene (12.5%) and *β*-caryophyllene (10.7%) as main compounds. Palmitic* *(26.5%) and nonadecanoic acids (26.6%) were determined as fatty acids. Phytochemical analysis of the extracts found phenolic acids, phlinosides, verbascoside, and flavonoids. The extracts and essential oil demonstrated poor *α*-amylase inhibitory activity. The best acetylcholinesterase inhibitory activity was obtained for diethly ether extract of *P. linearis* (67.2 ± 3.4%) at 10 mg /mL concentration. Ethyl acetate extract found to be effective against *Staphlococcus aureus* at a minimum inhibitory concentration (MIC) of 156.26 µg/mL. Diethyl ether extract of *P. linearis *was active on A549 cell lines with an IC_50_ = 316 ± 4.16 µg/mL when compared with cisplatin IC_50_ = 24.43 ± 0.14 µg/mL. To the best of our knowledge, the present work is the first comprehensive report on anti-acetylcholinesterase, anti-α-amylase, and antimicrobial activities, as well as cytotoxic effects of *P. linearis*.

## 1. Introduction

Medicinal plants have been very popular with researchers for the investigation of chemical profile and biological properties. Phytochemicals present in plants are valuable compounds of the human diet and used for the prevention of chronic diseases, such as degenerative disorders, cancer and diabetes, and as antiinfective agents. Plant secondary metabolites have many biological properties as they are antimicrobial [1–2], antioxidant, [3], enzyme inhibitor [4], antiinflammatory [5], antiproliferative [6], anticancer [7], and antidiabetic [8]. The antimicrobial activity of plant extracts and essential oils (EOs) has been known for many years. The researchers have documented various publications with the antimicrobial activity of Eos and plant extracts [2,9–12], their cytotoxic [7,13], and management of diabetes as α-amylase inhibitors [14–17].

The genus *Phlomis* L. (Lamiaceae) includes over 100 species and originates from Turkey, North Africa, Europe, and Asia.* Phlomis* species are known as “Ballıkotu, calba, çalba, şalba” and used generally for gastro-protective, hepatoprotective activities, and cardiovascular system disorders. The endemic species *Phlomis linearis* Boiss. & Bal. grow in central, east, and southeast Anatolia in Turkey. It is known as “Yaylaotu” in Turkish traditional medicine and consumed as herbal tea in Turkey for carminative and stimulating effects [18]. *P. linearis* has scarcely investigated for phytochemical properties. Iridoids and phenylethanoid type glycosides [19­–21] and EO compositions [22] have been reported earlier. Antiangiogenic and antiinflammatory activities or the EO of *P. linearis* has been investigated by Demirci et al. [22] on the chorioallantoic membrane.

To date, very little research has been carried out on phytochemistry and biological activity for *P. linearis*; there have been no attempts to examine the anti-acetylcholinesterase, anti-α-amylase, and antimicrobial activities, as well as cytotoxic effects against human lung cancer, human colon cancer, and mouse embryonic fibroblast cell lines for *P. linearis*. We aimed to investigate the phytochemical characterisation of EO, fatty acids and extracts from aerial parts of *P. linearis*, and biological activities of the extracts and EO.

## 2. Materials and methods

### 2.1. Chemicals

The chemicals* n-*Hexane (H), diethyl ether (DE) ethyl acetate (EAc), methanol (MeOH), dimethyl sulfoxide (DMSO) used of analytical grade. The enzyme *α*-amylase from porcine pancreas (type VI-B), acarbose, galanthamine from *Lycoris* sp., and acetylcholinesterase (AChE) from *Electrophorus electricus* (200–1000 units/mg protein, type VI-S) were obtained from Sigma–Aldrich Corp. (St. Louis, MO, USA). Acetylthiocholine iodide (ATCI) were purchased from Sigma-Aldrich Corp. Microorganisms were obtained from Microbiologics (San Diego, CA). Boron trifluoride reagent (BF3) solution in MeOH purchased from (Sigma–Aldrich Corp., city?, Germany). The reference solution (C_8_–C_40_ *n*-alkane series) was obtained from Fluka (Fluka Buchs, Switzerland).

### 2.2. Plant material

Plant material of *Phlomis linearis *was collected in Sivas province of Turkey: Çamlıbel 1850 m, 39o5801,5” N, 36o3253,8” E on* *July 03, 2019. The plant material was identified and deposited at Trakya University, Faculty of Pharmacy. Voucher number was given by M. Tekin (1822).

### 2.3. Essential oil isolation

Plant material of *P. linearis* (40 g) was exposed to hydrodistillation (3 h) in the Clevenger apparatus to yield EO [23]. The EO yield was calculated based on dried plant material and stored in an amber vial at 4 °C up until the phytochemical and biological activity analyses.

### 2.4. Preparation of extracts 

Plant material of *P. linearis *(40 g) were* *subjected to maceration with *n*-hexane, diethyl ether, ethyl acetate, and methanol (200 mL × 3), respectively for 24 h. The resulting extracts were collected, filtered, and concentrated in a rotary evaporator. The dried extracts were kept at 4°C.

### 2.5. Fatty acids analysis

The lipid extraction kit is used for the extraction of the total lipids from *P. linearis.* According to protocol, 0.15 g mill-ground plant material was treated with a 3 mL solvent containing chloroform/MeOH (2:1). After homogenising and vortexing of mixture, 0.5 mL of an aqueous buffer of the kit was added, and the sample was mixed by a vortex again. Subsequently, the extraction solution was poured into a syringe system containing a filter. The eluted solvent contained the chloroform phase with total lipids. Then, 200 mL of aliquot of the total lipids dried under a stream of nitrogen for subsequent transesterification. After drying, 1 mL of BF_3_-MeOH solution and 0.3 mL of *n*-hexane were added. The mixture was heated at 95°C for 1 h under reflux. Then, 1 mL of *n*-hexane and 1 mL of distilled water were added. The mixture was vortexed and centrifuged at 500 × g for 5 min. The top hexane layer was transferred into a vial and then injected into the GC-MS and GC-FID system without solvent evaporation before injection.

#### 2.6. Gas chromatographic analysis

GC-MS analysis was examined by an Agilent 6890N GC and Agilent 5975 GC/MSD systems (Agilent Technologies, SEM Ltd., Istanbul, Turkey). HP-Innowax FSC column (60 m × 0.25 mm, 0.25 μm film thickness (Agilent Technologies) was used with a helium carrier gas at 0.8 mL/min as reported previously [24].

##### 2.6.1. Identification of compounds

The compounds were identified by comparison of their mass spectra with those in Wiley NIST Library (NY, USA), Mass Finder software 4.0 (Dr. Hochmuth Scientific Consulting, Hamburg, Germany), and Adams Library (digital library)The National Institute of Standards and Technology (NIST) Chemistry WebBook, SRD 69 [2018] [online]. Website https://webbook.nist.gov/chemistry/ [accesed 05 September 2020]. Hochmuth, K., W.A. König, and D. Julain, MassFinder 4 software tool [online]. [Website] (http://massfinder.com/wiki/MassFinder_4) [accesed 05 September 2020].. In addition, identification of compounds was confirmed by comparison of their RRI values with data reported for polar column. The relative percentage amounts of the separated compounds were determined from FID chromatograms. Confirmation was done using the in-house “Başer Library of Essential Oil Constituents” database, analysed with known pure compounds by chromatographic runs at the same conditions.

### 2.7. LC- MS/MS analysis 

LC-MS/MS analyses of extracts were carried out using the Applied Biosystems 3200 Q-Trap LC- MS/MS (Antes, İstanbul, Turkey) system equipped with an ESI source operating in negative ion mode. For the chromatographic separation, a GL Science Intersil ODS (Tokyo, Japan) 250 × 4.6 mm, i.d., 5 µm particle size, octadecyl silica gel analytical column operating at 40 °C was used. The solvent flow rate was maintained at 0.5 mL/min. Detection was carried out with PDA detector. The elution gradient consisted of mobile phases (A) acetonitrile:water:formic acid (10:89:1,v/v/v), and (B) acetonitrile:water:formic acid (89:10:1, v/v/v), respectively. The amount of B was increased from 10% to 100% in 40 min. LC-ESI-MS/MS data were collected, and processed by Analyst 1.6 software [25­–26]. 

### 2.8. Determination of α-amylase inhibition

The antidiabetic potential of *P. linearis* extracts and EO was determined upon inhibition of the *α*-amylase enzyme that is involved in hydrocarbon’s metabolism. The iodine/potassium iodide (I/KI) method was used [27]. The concentration of samples was prepared with MeOH at 10 mg/mL, and the enzyme was prepared with 0.8 U/mL in 20 mM in sodium phosphate buffer pH (6.9). The control wells contained all the reagents without the sample (the solvents of the samples instead were added). Acarbose (inhibitor of α-amylase) prepared in concentration of 0.25 mg/mL was used as the positive control. The percentage of inhibition was calculated according to Equation (1):

Inhibition %=(AbscontrAbscontr blank)-(Abssample-Abssample blank)(Abscontr-Abscontr blank)x100

*Abs*_*control*_: the absorbance of control; *Abs*_*control blank*_ : the absorbance of blank 

*Abs*_*sample*_ the absorbance of sample; *Abs*_*sample blank *_: the absorbance of blank 

### 2.9. Determination of acetylcholinesterase inhibition

Acetylcholinesterase (AChE) inhibition of the extracts and EO were evaluated according to Ellman’s method [28] with a slight modification. 

A total of 25 µL of the sample, 50 µL of buffer, and 25 µL of AChE (0.22 U/mL) solution added into the 96 well (flat-bottom) plates and incubated for at 25 °C for 15 min. After that, 125 µL of Ellman’s reagent DTNB (5,5-dithio-bis-(2-nitrobenzoic acid) (3.0 mM) and 25 µL of substrate ATCI (15 mM) were added. The mixture was programmed to stand at 25 °C for 15 min and read at 412 nm by a microplate reader (Biotek Powerwave XS, USA). Galanthamine solution was prepared at a concentration of 0.6 mg/mL and used as a positive control. Similarly, a blank control was prepared by adding the sample solution to all reaction reagents and added 25 µL of the buffer instead of the enzyme. The control wells contained all the reagents without the sample. The percentage of inhibition was calculated according to Equation (1). The mean standard error (± SEM) was used for evaluation of the data.

### 2.10. Minimum inhibitory concentration (MIC, µg/mL)

#### 2.10.1. Microbial strains 

The antimicrobial activity of the extracts was evaluated against pathogen microorganisms namely: *Escherichia coli* ATCC 8739, *Salmonella enterica *ATCC 14028, *Staphylococcus aureus* ATCC 6538, *Bacillus subtilis *subsp*. spizizeni* ATCC 6633 and *Candida albicans* ATCC 10231 by the microdilution method as reported in our previous works [2,25]. 

#### 2.10.2. Minimum inhibitory concentration (MIC, µg/mL)

Antimicrobial activity performed for different extracts of *P. linearis. *The extracts were prepared within DMSO, and the standard antimicrobial powders were obtained from Sanovel Pharmaceutical Industry (İstanbul, Turkey). Ampicillin, cefuroxime, and fluconazole were used as reference drugs. The MIC values of the strains were evaluated with a slight modification of microdilution methods [29­–30]. The extracts were diluted 2-fold initially with a final concentration range of 2500 to 19.53 µg/mL. Ampicillin, cefuroxime, and fluconazole prepared at 64–0.125 µg/mL within DMSO and water. Bacterial suspensions were grown overnight in double strength broth and standardized to 10^5^ CFU/mL for bacteria. *Candida* suspensions were standardized using a turbidimeter (McFarland densitometer, Biosan, Latvia, 0.5 density) to 5 x 10^3^ CFU per well in RPMI medium under sterile conditions. 10 μL yeast inoculum was added to each well of the microplates.

After serial dilution of samples in 96 well, each microorganism suspension was pipetted into each well and incubated at 35 °C for 24 h. Positive growth controls (to assess the presence of turbidity) were performed in wells not containing antimicrobial agents. Microbial growth was observed by adding 20 µL of resazurin of 0.01% with minor modifications of CLSI standards. The experiment was done in triplicate and calculated the mean of MIC.

### 2.11. In vitro cytotoxicity assay 

Human lung epithelial cell line (A549, ATCC CCL-185), human colon cancer cell line (HT-29, ATCC® HTB-38), and mouse embryonic fibroblast cell line (NIH/3T3, ATCC CRL-1658) were used for determining IC_50_ of the extracts by MTT method according to previous studies [31–33]. Stock solutions of the extracts were prepared in ethanol. The tested extracts were added to the wells (1000–7.8125 µg/mL) in quadruplicates. Inhibition % was calculated for the extracts. Nonlinear regression analysis was used for IC_50_ values. Calculations on the results were performed according to Equation (2):

Inhibition %=100-(ODtest compound-ODblank)(ODsolvent control-ODblank)x100

#### 2.11.1 Selectivity index

Selectivity index (SI) was also calculated to compare the selectivity of the compounds according to a previous study [34] as follows:

SI = IC_50_ of compound in the NIH3T3 cells / IC_50_ of the same compound in the cancer cells.

## 3. Results and discussion

### 3.1. Chemical composition of essential oil

In the present study, we evaluated the chemical composition of the EO of *P. linearis* obtained by hydrodistillation of aerial part of *P. linearis*. The hydrodisitillation resulted with yellowish EO with a pleasant odour. The oil yield calculated on water free basis was 0.07% (w/v). Chemical composition of EO was analysed with GC-MS/FID systems. The list of volatile compounds determined in the EO of *P. linearis* with their relative retention indices, relative percentages are shown in Table 1. Twenty-four compounds were determined in the EO, representing 97.0% of the total oil composition. The major compounds of the oil were presented by monoterpene *α*-pinene (12.5%) and sesquiterpenes namely: *β*-caryophyllene (10.7%), *α*-cadinol (10.4%), and germacrene D (8.8%). The major representatives of the oxygenated sesquiterpenes (32.8%) were found to be as *α-*cadinol (10.4%), *α*-eudesmol (5.7%), caryophyllene oxide (5.1%), *τ*-muurolol (4.2%), *β*-eudesmol (4.0%), and *δ*-cadinol (3.4%). The sesquiterpene hydrocarbons (28.2 %) were the second important group in the oil with *β*-caryophyllene (10.7%), germacrene D (8.8%), *δ*-cadinene (6.2 %) and *γ*-cadinene (2.5 %) as major constituents, respectively.

**Table 1:** Chemical composition of Phlomis linearis essential oil.

No	RRI Lit.	Compound	%
1	1032 [35]	a-pinene	12.5
2	1203 [35]	limonene	2.4
3	1528 [35]	a-bourbonene	t
4	1535 [35]	b-bourbonene	1.9
5	1612 [35]	b-caryophyllene	10.7
6	1668 [35]	(Z)-b-farnesene	1.4
7	1671 [35]	acetophenone	7.5
8	1687 [35]	a-humulene	1.3
9	1704 [35]	g-muurolene	0.8
10	1726 [35]	germacrene D	8.8
11	1740 [35]	a-muurolene	1.5
12	1773 [35]	d-cadinene	6.2
13	1776 [35]	g-cadinene	2.5
14	1900 [35]	epi-cubebol	0.6
15	1957 [35]	cubebol	1.7
16	2008 [35]	caryophyllene oxide	5.1
17	2069 [36]	1,6-germacradien-5b-ol (1(10),5-germacradien-4b-ol)	1.2
18	2125 [35]	hexahydro-farnesylacetone	1.9
19	2187 [37]	τ- cadinol	3.4
20	2209 [38]	τ -muurolol	4.2
21	2219 [35]	d-cadinol ( = α-muurolol; torreyol)	1.3
22	2250 [35]	a-eudesmol	5.7
23	2255 [35]	a-cadinol	10.4
24	2257 [35]	b-eudesmol	4.0
		Total	97.0

RRI: Relative retention indices calculated against n-alkanes.

Earlier, Demirci et. al. [22] reported that the oil of *P. linearis* was identified with *β*-caryophyllene (24.2%), germacrene D (22.3%), and caryophyllene oxide (9.2%). 

The sample collected by us in Sivas province was characterised with quit rather content of monoterpenes (14.9%) with predominance of *α*-pinene (12.5%). However, the plant sample collected in Kayseri province did not contain monoterpenes at all. In addition, acetophenone was also not mentioned in the previous report [22]. High percentage of acetophenone (7.5%) was found in EO from fresh leaves of *P. umbrosa *Turcz. [39]. 

Observation of the literature showed that *α*-pinene is the main compound in different *Phlomis* species. Namely,* α*-pinene (39-57%) was found as a main compund of EO of *P. fruticosa* L. from two different localities in Yugoslavia [40]. High percentage of *α*-pinene (11.2%) was reported for *P. cretica* C. Presl [41] *P. lanata* Willd. (25.41%) [42] and *P. olivieri *Benth. (11.7%) [43]. 

### 3.2. Fatty acids compositions

The total lipids of *P. linearis* were obtained with microextraction technique with subsequent transesterification of fatty acids. Gas-chromatographic analysis gave nine compounds representing 97.4% of fatty acids (Table 2). The fatty acids fraction of *P. linearis* was characterised with abundance of saturated fatty acids. Namely, palmitic (26.5%), nonadecanoic (26.5%), and stearic (10.6%) acids were found as major fatty acids. In literature, there are several reports about fatty acid composition of *Phlomis* species. Namely, *P. bracteosa* Royle ex Benth. was characterised with octadecadienoic (6.8%), elaidic (4.4%), pentadecanoic (3.8%), and stearic (1.9%) acids [44]. Palmitic (33.1%, 27.4%, 27.8%), *α*-linolenic (23.1%, 24.4%, 24.6%), and oleic (10.5%, 23.7%, 14.4%) acids have been reported for *P. armeniaca *Willd.,* P. nissolii *L., and* P. pungens *var.* pungens *Willd., respectively [45]. Hexadecanoic acid in *P. herba-venti *L. leaves (12.9%) and flowers (33.1%) [46], octadecanoic acid in *P. bruguieri* Desf. (56.41%), and *P. olivieri* (44.4%) have been detected [47]. To best of our knowledge, the fatty acid composition of *P. linearis *have not previously been reported. Therefore, the current study is the first report on lipophilic constituents of* P. linearis.*

**Table 2:** Fatty acids compositions of Phlomis linearis.

No	RRI	Compound	%
1	1810	methyl dodecanoate (methyl laurate)	3.3
2	2018	methyl tetradecanoate (methyl myristate)	2.1
3	2223	methyl hexadecanoate (methyl palmitate)	26.5
4	2431	methyl octadecanoate (methyl stearate)	10.6
5	2468	(Z)-9-methyl octadecanoate (methyl oleate)	5.0
6	2509	(Z,Z)-9,12-methyl octadecadienoate (methyl linoleate)	8.4
7	2526	methyl nonadecanoate	26.5
8	2572	methyl linolenate	9.8
9	2841	methyl behenate (methyl docosanoate)	5.2
		Total	97.4

### 3.3. Ethyl acetate and methanol extracts composition of P. linearis 

**Figure 1:**
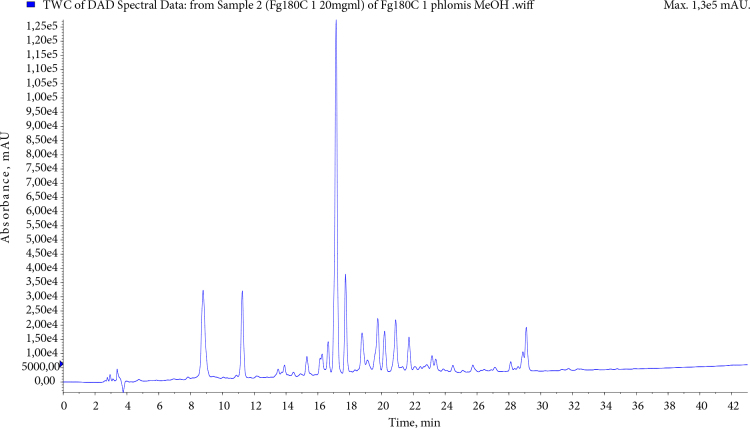
HPLC chromatogram of P. linearis MeOH extract.

**Figure 2:**
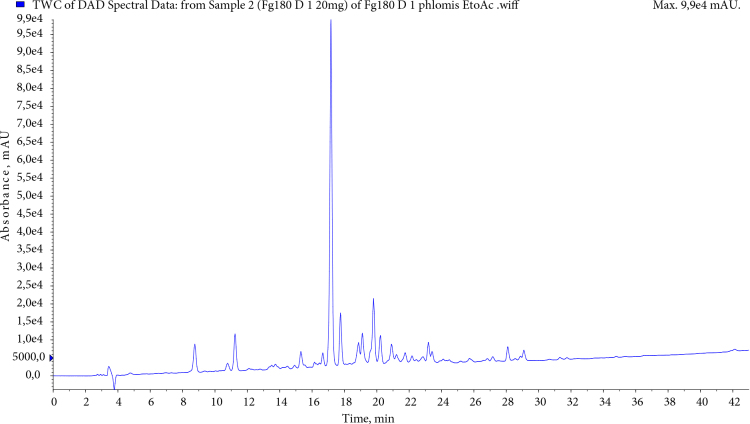
HPLC chromatogram of P. linearis EAc extract.

Phenolics acids, flavonoids, and phenylethanoid glycosides were identified for the ethyl acetate and methanol extracts via LC-MS/MS technique. The list of the compounds detected in *P. linearis* ethyl acetate and methanol extract with MS detector is summarised in Table 3. The results of phytochemical analyses of the extracts were examined with 5-caffeoylquinic acid, 3.5/1.5 dicaffeoylquinic acid, phlinosides, verbascoside, quercetin, and luteolin derivatives. Chromatographic profiles EAc and MeOH extracts of *P. linearis* obtained with liquid chromatography were given in Figures 1 and 2. 

**Table 3:** LC-MS/MS analysis of the extracts.

RT	[M-H]+	MS2	Identified as	Extracts	Reference
8.6	467	421, 403, 385, 331, 179	unknown	M, EAc	-
11.4	353	191, 179, 173, 135	5-caffeoylquinic acid	M, EAc	[48–49]
12.8	401	269, 161	apigenin pentoside	M, EAc	[50]
14.0	593	503, 473, 383, 353, 325, 297	apigenin 6,8-C-diglucoside	M, EAc	[51]
15.0	273	211, 183, 167, 141,133	unknown	M, EAc	-
16.4	755	593, 461, 179, 161	phlinoside B	M, EAc	[19]
17.9	785	623, 461, 161	phlinosides A	M, EAc	[19]
17.1	755	623, 593, 461, 179, 161	forsythoside B	M, EAc	[48,52]
17.3	623	461, 315, 297, 179, 161, 135	verbascoside (main compound)	M, EAc	[20,52]
17.8	769	623, 607, 461, 315, 297	phlinosides C/D	M, EAc	[19­–20]
18.8	595	463, 343, 300, 271, 255	quercetin glucoside + pentoside	M, EAc	[53]
19.1	515	353, 191, 179,	3,5 /1,5 dicaffeoylquinic acid	M, EAc	[54]
20.0	637	461, 300, 193, 175	leukoptoside A	M, EAc	[20,52]
20.3	447	285	luteolin glucoside	M, EAc	[55]
21.2	463	301, 271, 255	quercetin glucoside	M, EAc	[55]
21.7	505	300, 271, 255, 179, 151	quercetin acetylglucoside	M, EAc	[56]
23.3	461	446, 323, 300, 161, 137	chrysoeriol glucoside	M, EAc	[48,57]
23.3	651	475, 175	martynoside	M, EAc	[20,52]
23.8	477	314, 299, 285, 271,	isorhamnetin glucoside	M, EAc	[65]
24.6	489	285	luteolin acetylglucoside	M, EAc	[58]
25.8	609	463, 300, 271, 255	quercetin rutinoside	M, EAc	[59]
27.2	593	285	luteolin rutinoside	M, EAc	[55]
27.3	271	151	naringenin	M, EAc	[55]
28.6	285	151, 133	luteolin	M, EAc	[55,57]
29.1	607	461, 300, 284	chrysoeriol rutinoside	M, EAc	[55]

M: methanol; EAc: ethyl acetate extract. The column: GL Science Intersil ODS (250 × 4.6 mm, i.d., 5 µm particle size)

### 3.4. α-Amylase inhibitory activity 

Diabetes mellitus is a metabolic disorder and characterised by hyperglycemia. α-amylase is a key enzyme that hydrolysis carbohydrates to disaccharides, and α-glucosidases hydrolysis disaccharides to monosaccharides like glucose. Therefore, inhibition of these enzyme systems helps to control hyperglycemia and digestion of carbohydrates to reduce blood glucose levels [60–61].

In this study, the extracts and EO of *P. linearis* were evaluated for in vitro *α*-amylase activity. As indicated in Table 4, the EO inhibited the enzyme’s activity by 25.7% at concentration of 10 mg/mL. The following order of the extracts against α-amylase activity was observed at 10 mg/mL concentration: *n*-hexane (31.5 ± 2.6%), methanol (30.5 ± 1.4), diethyl ether (28.3 ± 4.0%) and ethyl acetate (24.8 ± 2.0%). It should be noted that acarbose displayed more potent inhibition of α-amylase (57.2%, concentration at 0.25 mg/mL) than all tested extracts and EO.

**Table 4:** Acetylcholinesterase and α-amylase inhibitory (%) activities of P. linearis

Extracts/standards	AChE (% Inh)	α-amilaz (% Inh)
PL-H	42.8 ± 5.8	31.5 ± 2.6
PL-DE	67.2 ± 3.4	28.3 ± 4.0
PL-EAc	9.2 ± 3.5	24.8 ± 2.0
PL-MeOH	44.7 ± 1.0	30.5 ± 1.4
PL- EO	39.5 ± 0.8	25.7 ± 2.0
Acarbose	-	57.2 ± 1.8
Galanthamine	71.3 ± 0.4	-

*PL-H, PL-DE, PL-EAc, PL-MeOH: hexane, diethyl ether, ethyl acetate, and methanol extracts of Phlomis linearis, respectively. PL-EO: essential oil of P.linearis

In this study, phenolics acids, flavonoids, and phenylethanoid glycosides were identified for the EAc and MeOH extracts. These compounds have been reported to have antidiabetic effects [62–64]. Twenty-one flavonoids were investigated for inhibitory activities against α-glucosidase and α-amylase. Luteolin inhibited α-glucosidase (36%, at 0.5 mg/mL) [63]. Six group of flavonoids were reported inhibitory activities against *α*-glucosidase and α-amylase. Among them, luteolin, myrcetin, and quercetin were found as potent inhibitors with IC_50_ of 0.36, 0.38, and 0.50 mM, respectively against *α*-amylase enzyme [64]. Moreover, MeOH extract of *Phlomis stewartii *displayed *α*-glucosidase inhibitory activity (80.2% at 1.0 mg/mL) [65]. The *α*-amylase inhibitory capacities of these extracts might be attributed to their phytochemicals contents.

However, there have been no reports for EO and different extracts of *P. linearis *against *α*-amylase inhibitory activity. The *α*-amylase inhibitory activity of the extracts *P. linearis* was performed for the first time in this study.

### 3.5. Acetylcholinesterase inhibitory activity 

The extracts and EO of *P. linearis* were in vitro evaluated for acetylcholinesterase enzyme inhibitory activity. AChE inhibition activity was represented as inhibition percentage and compared with galanthamine (Table 4).

The EO inhibited AChE (39.5 %) at 10 mg/mL concentration. In this report, EOs of *P.* *linearis* consist of *α*-pinene (12.5%) and *β*-caryophyllene (10.7%) as major compounds. In a previous research, α-pinene, 1,8-cineole, and camphor were found to be uncompetitive reversible inhibitors of AChE [66]. According to recent study, the EO of *P. kurdica* was also reported for AChE (41.4%) and butyrylcholinesterase (BChE) (36.2%) inhibitory activity at 250 µg/mL concentration [67]. Additionally, EOs for *Piper* species demonstrated their acetylcholinesterase activities. EOs contain terpenes and phenylpropanoids as predominant compounds like *P. linearis* essential oil. [68]. Therefore, anti-AChE activity of the tested *P. linearis *EOs could be attributed the presence of the predominant compounds. However, it should be noted that essential oils are mixture of many chemical compounds, which may potentially modulate enzyme inhibitions. Therefore, main and minor compounds of the EO may attempt to enzyme inhibitions.

The extracts of *P. linearis* demonstrated anti-AChE activity ranged between 9.2% and 67.2% at 10 mg/mL concentrations. The best inhibitory activity was obtained for DE extract of *P. linearis* (67.2%) and followed with methanol (44.7%) and hexane (42.8%) extracts. The ethyl acetate extract demonstrated poor inhibitory activity (9.2%). 

In this report, analysis of the extracts contains caffeoylquinic acid and its derivatives as well as flavonoids and phenylethanoid glycosides by LC-MS/MS. 

Previously a study reported anticholinesterase potentials of phenolic acids and various flavonoid derivatives [69]. In the current study, quercetin showed a considerable inhibition (76.2%) against AChE, while genistein (65.7%), luteolin-7-O-rutinoside (54.9%), and silibinin (51.4%) performed a moderate inhibition on BChE.

For this purpose, the AChE inhibitory activity of *P. linearis* extracts and essential oil has never been reported before, and obtained results indicate that *P. linearis* could serve as an inhibitor against AChE enzyme. 

### 3.6. Antimicrobial activity (MIC, µg/mL)

In this study, antimicrobial activity was evaluated for different extracts of *P. linearis* against *E. coli* ATCC 8739, *S. enterica* ATCC 14028, *B.subtilis *subsp* spizizeni *ATCC 6633, *S. aureus* ATCC 6538, and *C. albicans* ATCC 10231. Antimicrobial activity of the extracts was compared to cefuroxime, ampicillin, and fluconazole as the standard drugs as given in Table 5 by microdilution method.

**Table 5:** Minimum inhibitory concentrations (MIC = µg/mL).Extracts /standardsE. coli ATCC 8739S. enterica ATCC 14028B.subtilis subsp spizizeni ATCC 6633S. aureus ATCC 6538C. albicans ATCC 10231PL-H625625625312.5625PL-DE6251250625312.5625PL- EAc6251250312.5156.25625PL-MeOH625625625312.5625Vefuroxime4882 >-Ampicillin2 >2 >2 >2 >-Fluconazole----> 64* PL-H, PL-DE, PL-EAc, PL-MeOH: hexane, diethyl ether, ethyl acetate, and methanol extracts of Phlomis linearis, respectively.

Extracts /standards	E. coli ATCC 8739	S. enterica ATCC 14028	B.subtilis subsp spizizeni ATCC 6633	S. aureus ATCC 6538	C. albicans ATCC 10231
PL-H	625	625	625	312.5	625
PL-DE	625	1250	625	312.5	625
PL- EAc	625	1250	312.5	156.25	625
PL-MeOH	625	625	625	312.5	625
Vefuroxime	4	8	8	2 >	-
Ampicillin	2 >	2 >	2 >	2 >	-
Fluconazole	-	-	-	-	> 64

The most effective extract was found to be EtOAc extract against *S.aureus* ATCC 6538 strain with MIC value 156.25 µg/mL. All of the extracts had the same MIC values with 625 μg/mL against *E. coli* ATCC 8739 and *C. albicans* ATCC 10231 strains. All of the extracts generally were found to be most effective against S. aureus ATCC 6538 range of MIC = 156.25–312.5 μg/mL in our study.

Generally, antimicrobial acitivity have been focused on EO extracted from *Phlomis* species in the literature. The EOs of *P. ferruginea* Ten [70] *P. bovei* De Noe subsp. bovei [71] *P. bracteosa* Royle ex Benth. [72] *P. floccosa* D. Don [73], *P. kurdica* Rech. fil. [67] and isolated a few compounds as, forsythoside B, phlinoside C and verbascoside from *P. lanceolata* [74] showed antimicrobial activities, while the EO of *P. linearis* has been reported only in antiangiogenic and antiinflammatory activity [22]. In addition to EO, methanol extracts of *P. olivieri, P. bruguieri*,* and P.herba-venti* were investigated in terms of their antibacterial effects against some bacteria pathogens [46]. To the best of our knowledge, this paper represents the first report on the antimicrobial activities of *P. linearis* Boiss. & Bal on different extracts.

### 3.7. Cytotoxicity assay

MTT test was evaluated to see cytotoxic activity of the extracts of *P. linearis* against A549 and HT-29 cancer cell lines. Also, the cytotoxic activities of extracts were studied against NIH3T3 cells, and to determine the selectivity of the extracts towards carcinogenic cell lines. The IC_50_ values of the extracts were determined against cell lines in Table 6.

**Table 6:** Cytotoxic activitiy of the extracts of P. linearis.

Extracts /standards	Cell lines IC_50_ (µg/mL)			Selectivity index (SI)	
	NIH/3T3	A549	HT29	A549	HT29
PL-H	>1000	>1000	>1000	NC	NC
PL-DE	849.25 ± 29.81	316 ± 4.16	>1000	2.69	NC
PL-EAc	99.15 ± 3.75	316 ± 4.16	444.90 ± 16.39	0.31	0.22
PL-MeOH	613.52 ± 12.79	>1000	>1000	NC	NC
Cisplatin	ND	24.43 ± 0.14	216 ± 2.74	NC	NC

PL-H, PL-DE, PL-EAc, PL-MeOH: hexane, diethyl ether, ethyl acetate, and methanol extracts of Phlomis linearis, respectively. NC: not calculated. ND: not determined.

The compounds should be nontoxic on healthy cell lines and show cytotoxic effect in cancer cell lines as anticancer drug candidates. Hence, cytotoxic effect of the extracts against NIH3T3 cell line was tested. Cytotoxic activity was found to be EAc (IC_50_ = 99.15 µg/mL), MeOH (IC_50_ = 613.52 µg/mL), DE (IC_50_ = 849.25 µg/mL), and H (IC_50_ > 1000 µg/mL) extracts, respectively against NIH3T3 cells. EAc extract showed higher cytotoxicity on NIH3T3 cell lines. Conversely, H and DE extracts showed lower cytotoxicity on NIH3T3 cells.

DE (IC_50_ = 316 µg/mL) and EAc extract (IC_50_ = 316 µg/mL) were the most active extracts against A549 cell lines, while EAc extract was only found to be most active (IC_50_ = 444.9 µg/mL) against HT-29 cancer cell lines. 

As a conclusion, DE extract indicated lower cytotoxic effect on NIH3T3 cells (IC_50_ = 849.25 µg/mL), while it showed higher value of IC_50_ = 316 µg/mL cytotoxic effect on A549 cell line and its selectivity index was calculated as 2.69. This finding improved the DE extract can be employed as a candidate in anticancer therapy. This extract can lead into *in vivo* studies for further therapeutic development.

On the basis of previous investigations, *Phlomis* species have shown cytotoxic activity against various cancer cell lines. Cytotoxic activity of *P. lanceolata *displayed against HT29, Caco2, T47D, and NIH3T3 cell lines. Petroleum ether extract was found to be the most active against all four cell lines [75]. In another study, cytotoxic activity of the 80% MeOH extracts fallowing namely, *P. kurdica*, *P. bruguieri, P. caucasica, P. olivieri, P. anisodontea*, and* P. persica *were assessed against on HepG2, MCF7, HT29, and A549 cancer and one normal cell lines MDBK [76]. The present results have indicated aqueous anticancer effects of the of *P. russeliana* extract against Caco-2 cell lines [77].

To date, *P. linearis* has not been investigated for any cytotoxic assay. To the best of our knowledge, this is the first report on cytotoxic activity of *P. linearis* against two cancer cell lines and one normal cell line by MTT assay.

## 4. Conclusion 

The present work is the first investigation on essential oil and extracts from aerial parts of *P. linearis *to include on anti-acetylcholinesterase, anti-α-amylase, and antimicrobial activities, as well as cytotoxic effects. The phytochemical characterisation of essential oil, fatty acids, and extracts from aerial parts of *P. linearis* were represented using GC-MS /FID and LC/MS-MS techniques. The essential oils and extracts of *P. linearis* were found to have valuable phytochemicals with biological activities. The results showed that ethyl acetate extract of *P. linearis* possess high antibacterial activity against *S. aureus* ATCC 6538. Therefore, *P. linearis* could be recommended for the combination with antimicrobial drugs for drug industry, especially against *S. aureus.* Furthermore, diethyl ether extract indicated cytotoxic effect against human lung cancer cell lines. The best acetylcholinesterase inhibitory activity was obtained for diethyl ether extract of *P. linearis.*

Finally, *P. linearis *could be evaluated for isolation of active components for therapeutical applications. However, it needs further in vivo studies for safety and efficacy in the aforementioned applications.

**Acknowledgment**

This work was partially supported by the Scientific and Technological Research Council of Turkey (TÜBİTAK) with the project number SBAG 218S812).

## References

[ref1] (2013). Antioxidant and antimicrobial activities of essential oil and extracts of fennel (Foeniculum vulgare L.) and chamomile (Matricaria chamomilla L. Industrial Crops and Product.

[ref2] (2018). Antimicrobial and toxicity profiles evaluation of the chamomile (Matricaria recutita L.) essential oil combination with standard antimicrobial agents. Industrial Crops and Products.

[ref3] (2014). α-amylase inhibitory properties of different extracts from betel leaves. Industrial Crops and Produts.

[ref4] (2013). Assessment of cholinesterase and tyrosinase inhibitory and antioxidant effects of Hypericum perforatum L. (St. John’s wort). Industrial Crops and Products.

[ref5] (2007). A review of anti-infective and anti-inflammatory chalcones. European Journal of Medicinal Chemistry.

[ref6] (2011). Antiproliferation and induction of apoptosis by Moringa oleifera leaf extract on human cancer cells. Food and Chemical Toxicology.

[ref7] (2015). Medicinal plants: their use in anticancer treatment. International Journal of Pharmaceutical Sciences and Research.

[ref8] (2010). Antidiabetic activity of Vinca rosea extracts in alloxan-induced diabetic rats. International Journal of Endocrinology.

[ref9] (2002). Antimicrobial screening of Mentha piperita essential oils. Journal of Agricultural and Food Chemistry.

[ref10] (2009). Phytochemical screening and antimicrobial activity of the plant extracts of Mimosa pudica L. against selected microbes.

[ref11] (2010). and antioxidant efficacy of some medicinal plants against food borne pathogens. Advances in Biological Research.

[ref12] (2012). Antimicrobial activity of some important medicinal plant oils against human pathogens. Journal of Biologically Active Products from Nature.

[ref13] (2014). Essential oils of amazon Piper species and their cytotoxic, antifungal, antioxidant and anti-cholinesterase activities. Industrial Crops and Products.

[ref14] (2014). Evaluation of alpha-amylase inhibition by Urtica dioica and Juglans regia extracts. Iranian Journal of Basic Medical Sciences.

[ref15] (2016). In vitro alpha amylase inhibitory activity of the leaf extracts of Adenanthera pavonina. BMC Complementary and Alternative Medicine.

[ref16] (2019). Phytochemical profiling and evaluation of Marrubium sivasense Aytaç, Akgül & Ekici effects on oxidative damage, α-amylase, lipoxygenase, xanthine oxidase and tyrosinase enzymes. Journal of the Turkish Chemical Society Section A: Chemistry.

[ref17] (2019). Assessment of endemic Cota fulvida (Asteraceae) for phytochemical composition and inhibitory activities against oxidation, α-amylase, lipoxygenase, xanthine oxidase and tyrosinase Enzymes.

[ref18] (2016).

[ref19] (1990). B and C, three phenylpropanoid glycosides from Phlomis linearis. Phytochemistry.

[ref20] (1991). Phenylpropanoid glycosides, and iridoids from Phlomis linearis. Phytochemistry.

[ref21] (1991). Iridoid glucosides isolated from P. linearis. Hacettepe Üniversitesi Eczacılık Fakültesi Dergisi.

[ref22] (2003). Chemical composition of the essential oil of Phlomis linearis Boiss.

[ref23] (2005). Determination of essential oils in vegetable drugs. In: European Pharmacopea 5.0.

[ref24] (2018). Isolation of eudesmane type sesquiterpene ketone from Prangos heyniae H. Duman & MF Watson essential oil and mosquitocidal activity of the essential oils. Open Chemistry.

[ref25] (2015). Phytochemical characterization of phenolics by LC-MS/MS and biological evaluation of Ajuga orientalis from Turkey. Bangladesh Journal of Pharmacology.

[ref26] (2019). Two acylated isoscutellarein glucosides with anti-inflammatory and antioxidant activities isolated from endemic Stachys subnuda Montbret & Aucher ex Benth. Acta Chimica Slovenica.

[ref27] (2017). Composition and potential of Tanacetum haussknechtii Bornm. Grierson as antioxidant and inhibitor of acetylcholinesterase, tyrosinase and α-amylase enzymes. International Journal of Food Properties.

[ref28] (1961). A new and rapid colorimetric determination of acetylcholinesterase activity. Biochemical Pharmacology.

[ref29] (2006). Clinical and Laboratory Standarts Institue (CLSI). CLSI M7-A7.

[ref30] (2008). Clinical and Laboratory Standards Institute (CLSI). Reference Method for Broth Dilution Antifungal Susceptibility Testing of Yeast. Approved standard. CLSI 27-A3.

[ref31] (2018). Synthesis of new benzothiazole acylhydrazones as anticancer agents. Molecules.

[ref32] (2016). AChE inhibitory activity of new benzothiazole–piperazines. Bioorganic & Medicinal Chemistry Letters.

[ref33] (2016). Synthesis of new donepezil analogues and investigation of their effects on cholinesterase enzymes. European Journal of Medicinal Chemistry.

[ref34] (2010). In vitro cytotoxicity of benzopyranone derivatives with basic side chain against human lung cell lines. Anticancer Research.

[ref35] (2011). Retention indices for frequently reported compounds of plant essential oils. Journal of Physical and Chemical Reference Data.

[ref36] (2017). Chemical and biological diversity of the leaf and rhizome volatiles of Acorus calamus L. from Turkey. Journal of Essential Oil Bearing Plants.

[ref37] (2006). Essential oil analysis and anticancer activity of leaf essential oil of Croton flavens L. from Guadeloupe. Journal of Ethnopharmacology.

[ref38] (2004). Chemistry of the Australian gymnosperms. Biochemical Systematics and Ecology.

[ref39] (2008). Study on volatile components and biology activities of the essential oil from leaf of Phlomis umbrosa. Food Science and Technology International.

[ref40] (2000). Antimicrobial activity of essential oils and ethanol extract of Phlomis fruticosa L.(Lamiaceae). Phytotheraphy Research.

[ref41] (2006). The essential oil composition of Phlomis cretica C. Presl. Flavour and Fragrance Journal.

[ref42] (2002). Essential oil of Phlomis lanata growing in Greece: chemical composition and antimicrobial activity. Planta Medica.

[ref43] (2003). Volatile constituents of Phlomis olivieri Benth. from Iran. Flavour and Fragrance Journal.

[ref44] (2013). Analysis of the fatty acid composition of Phlomis bracteosa oil by gas chromatography mass spectrometer. Life Science Journal.

[ref45] (2016). Chemical composition, antioxidant, and enzyme inhibitory activities of the essential oils of three Phlomis species as well as their fatty acid compositions. Food Science and Biotechnology.

[ref46] (2004). The essential oils composition of Phlomis herba-venti L. leaves and flowers of Iranian origin. Flavour and Fragrance Journal.

[ref47] (2016). Analysis of fatty acid composition of two selected Phlomis species. Journal of Herbmed Pharmacology.

[ref48] (2004). Glycosides from Phlomis lunariifolia. Phytochemistry.

[ref49] (2003). Hierarchical scheme for LC-MSn identification of chlorogenic acids. Journal of Agricultural and Food Chemistry.

[ref50] (2013). Characterisation of phenolic compounds in wild fruits from Northeastern Portugal. Food Chemistry.

[ref51] (2011). Approach to the study of C-glycosyl flavones acylated with aliphatic and aromatic acids from Spergularia rubra by high-performance liquid chromatography-photodiode array detection/electrospray ionization multi-stage mass spectrometry. Rapid Communications in Mass Spectrometry.

[ref52] (2005). Identiﬁcation by HPLC-PAD-MS and quantiﬁcation by HPLC-PAD of phenylethanoid glycosides of five Phlomis species. Phytochemical Analysis: An International Journal of Plant Chemical and Biochemical Techniques.

[ref53] (2006). Structural characterization of flavonol 3, 7-di-O-glycosides and determination of the glycosylation position by using negative ion electrospray ionization tandem mass spectrometry. Journal of Mass Spectrometry.

[ref54] (2005). Discriminating between the six isomers of dicaffeoylquinic acid by LC-MS. Journal of Agricultural and Food Chemistry.

[ref55] (2018). Flavonoid constituents of Phlomis (Lamiaceae) species using liquid chromatography mass spectrometry. Phytochemical Analysis.

[ref56] (2015). Hydroxycinnamic acids and flavonols in native edible berries of South Patagonia. Food Chemistry.

[ref57] (2007). Flavonoids from Phlomis fruticosa (Lamiaceae) growing in Montenegro. Biochemical Systematics and Ecology.

[ref58] (2000). Negative atmospheric pressure chemical ionisation low-energy collision activation mass spectrometry for the characterisation of flavonoids in extracts of fresh herbs. Journal of Chromatography A.

[ref59] (2014). Polyphenols in representative Teucrium species in the Flora of R. Macedonia: LC/DAD/ESI-MS Profile and Content. Natural Product Communication.

[ref60] (2013). Mishra A. In vitro studies on alpha amylase and alpha glucosidase inhibitory activities of selected plant extracts. European Journal of Experimental Biology.

[ref61] (2017). -amylase/α-glucosidase inhibitory activities of plant-derived phenolic compounds: a virtual screening perspective for the treatment of obesity and diabetes. Food & Function.

[ref62] (2019). Two new glycosides from the leaves of Ligustrum robustum and their antioxidant activities and inhibitory effects on α-glucosidase and α-amylase.

[ref63] (2000). Inhibition of alpha-glucosidase and amylase by luteolin, a flavonoid. Bioscience.

[ref64] (2006). Inhibition of α-glucosidase and α-amylase by flavonoids. Journal of Nutritional Science and Vitaminology.

[ref65] (2013). Isolation of natural compounds from Phlomis stewartii showing α-glucosidase inhibitory activity. Phytochemistry.

[ref66] (2000). In vitro inhibition of human erythrocyte acetylcholinesterase by Salvia lavandulaefolia essential oil and constituent terpenes. Journal of Pharmacy and Pharmacology.

[ref67] (2020). Antimicrobial, anticholinesterase evaluation and chemical characterization of essential oil Phlomis kurdica Rech. fil. Journal of Essential Oil Research.

[ref68] (2017). Chemical composition and acetylcholinesterase inhibitory activity of essential oils from Piper species. Journal of Agricultural and Food Chemistry.

[ref69] (2007). Screening of various phenolic acids and flavonoid derivatives for their anticholinesterase potential.

[ref70] (2006). Chemical composition and antimicrobial activity of the essential oil of Phlomis ferruginea Ten. (Lamiaceae) growing wild in Southern Italy. Flavour and Fragrance Journal.

[ref71] (2007). Chemical composition and antimicrobial activity of the essential oil of Algerian Phlomis bovei De Noe subsp.

[ref72] (2011). Antimicrobial activity of the essential oil of Phlomis bracteosa. Scientific World.

[ref73] (2019). Chromatographic analysis, antimicrobial and insecticidal activities of the essential oil of Phlomis floccosa D.

[ref74] (2008). Assessment of the antibacterial activity of phenylethanoid glycosides from Phlomis lanceolata against multiple-drug-resistant strains of Staphylococcus aureus. Journal of Natural Medicines.

[ref75] (2014). Investigating the effect of Phlomis lanceolata Boiss. and hohen on cancer cell lines. Acta Medica Iranica.

[ref76] (2017). Evaluation of the cytotoxic activity of extracts from six species of Phlomis genus. Journal of Applied Pharmaceutical Sciences.

[ref77] (2019). Evaluating antimicrobial and antioxidant capacity of endemic Phlomis russeliana from Turkey and its antiproliferative effect on human Caco-2 cell lines.

